# Supplementary material for: Upregulation of Thioredoxin Reductase 1 Expression by Flavan-3-Ols Protects Human Kidney Proximal Tubular Cells from Hypoxia-Induced Cell Death
Source: Antioxidants (Basel). 2022 Jul 19;11(7):1399. doi: 10.3390/antiox11071399 (PMC9311547; doi:10.3390/antiox11071399)
Supplement: Supplementary file 1 [file antioxidants-11-01399-s001.zip › antioxidants-1734823-supplementary.pdf]

Suppl material: Figure S1

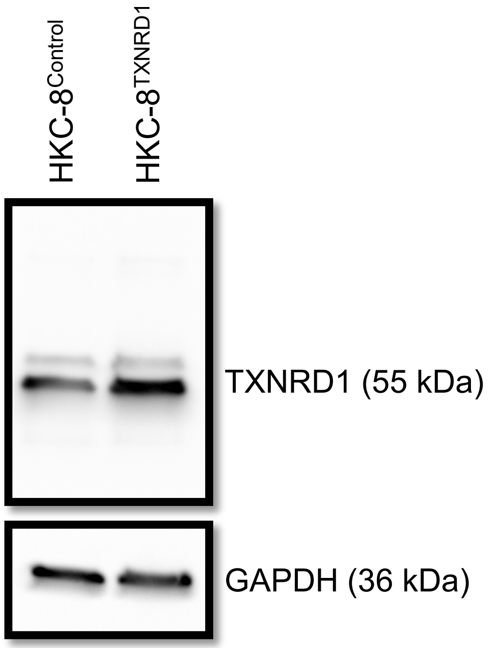

Figure S1. The protein levels of TXNRD1 in both HKC-8^{TXNRD1} and HKC-8^{Control} cells. Protein samples were prepared from cells that were grown in K1$^{+/+}$ medium in a CO_2 incubator at 37 ^{oC}. The levels of TXNRD1 protein were semi-quantitatively determined using Western blot analysis. Data are a representative of three separate experiments

Suppl material: Figure S2

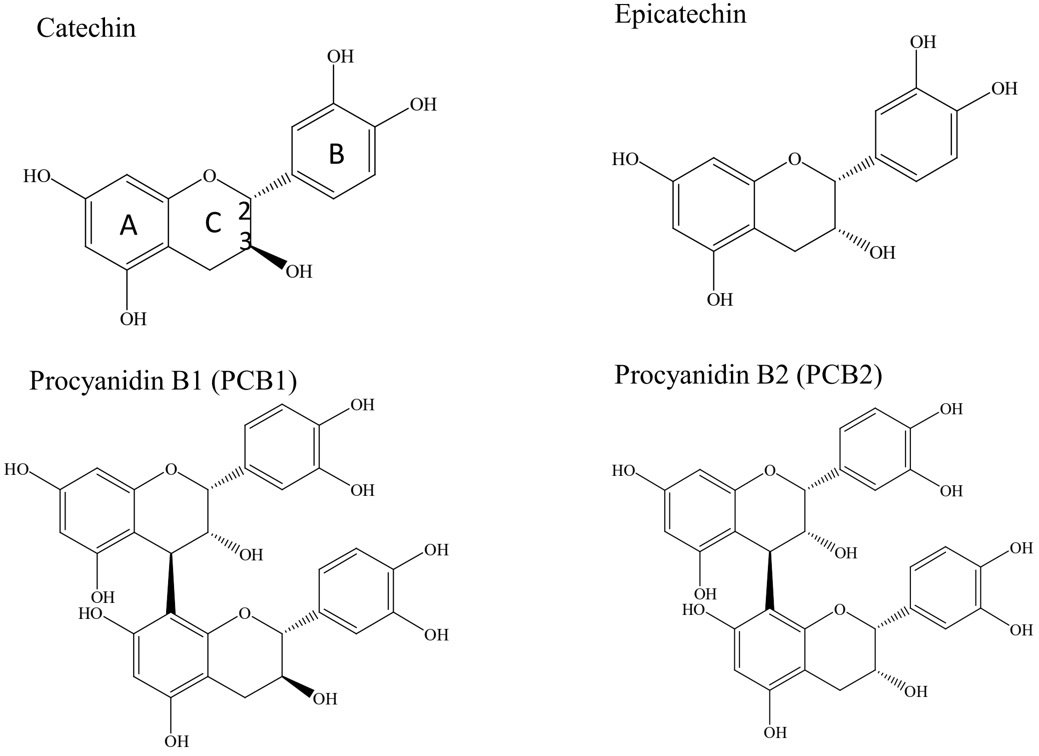

Figure S2. The chemical structures of catechin, epicatechin, PCB1, and PCB2. The common structure consists of A- and B-benzene rings and a dihydropyran heterocycle C-ring with a hydroxyl group on carbon 3. Two chiral centers on the structure on carbon 2 and 3. Stereoisomers are found in *trans* [(+)-catechin] or *cis* [(-)-epicatechin] configuration on carbon 2 and 3.

Suppl material: Figure S3

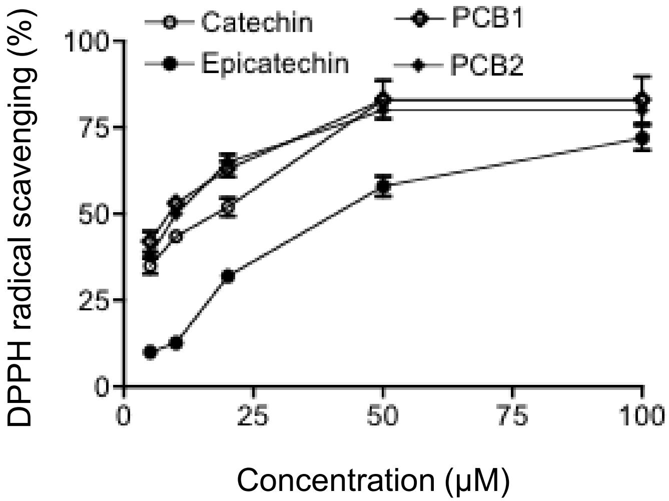

Figure S3. The DPPH (2,2-diphenyl-1-picrylhydrazyl) radical scavenging activities of catechin, epicatechin, PCB1, and PCB2. The optical density of 510 nm – the reaction of DPPH with different concentrations of flavon-3-ols was determined as described in our previous study (Reference 13). The positive control: 100 μM of L-Ascorbic acid that inhibited 78.5 ± 5% of DPPH. Data are presented as mean ± SD of three determinants.

Suppl material: Figure S4

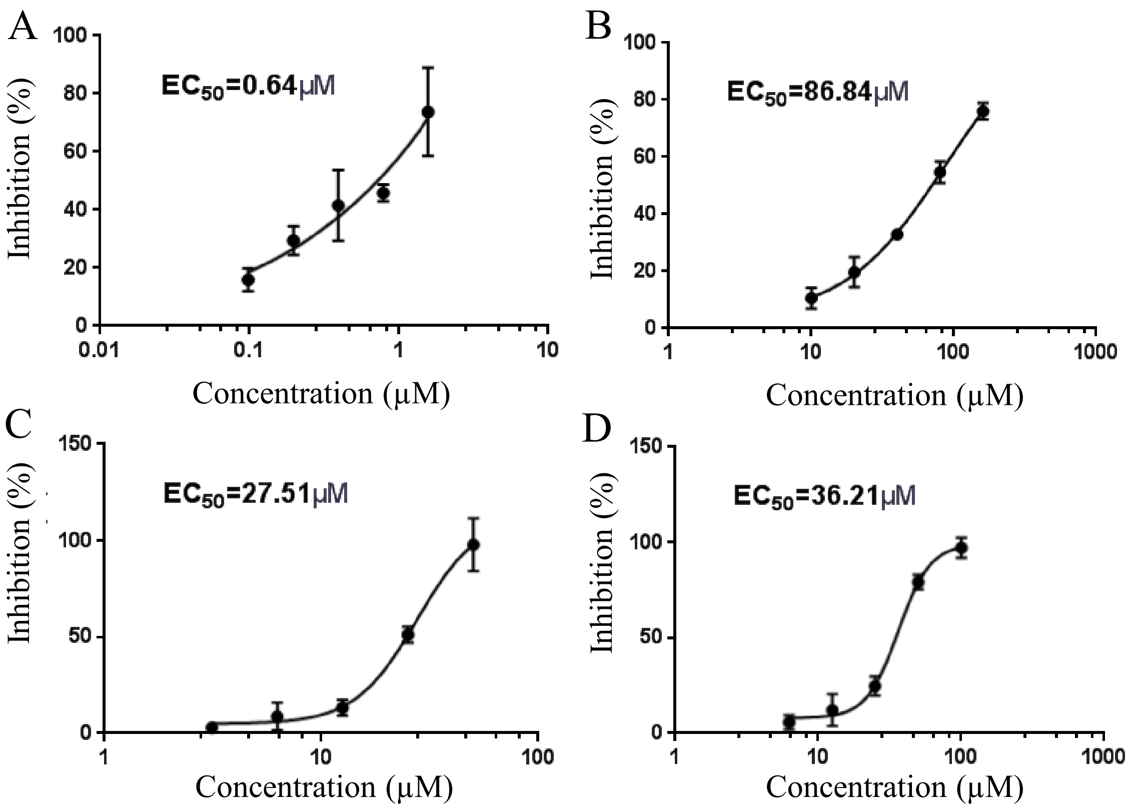

Figure S4. Different effects of flavan-3-ols on H_2O_2-induced cell death in HKC-8 cells. HKC-8 cells in 96-well plates were treated with 0.5 µM H_2O_2 in the absence or presence of flavan-3-ols at different concentrations for 48 h. Cell viability was measured using MTT assay. Percentage inhibition of cell death (reduced optical density – OD) by flavan-3-ols was determined using the formula $(T - F)/T \times 100$, where T was cell death by H_2O_2 without treatment, and F was the cell death in cultures treated with flavan-3-ols, respectively. The EC_{50} value of each flavan-3-ol was calculated based on the inhibition curve. A) Catechin, B) Epicatechin, C) PCB1, D) PCB2. Data are presented as mean ± standard deviation of 4 determinants and are a representative of three separate experiments

Table S1. A gene list for PCR array analysis of oxidative stress in HKC-8 cells

Number	UniGene	Genbank	Symbol	Description
1	Hs.418167	NM_000477	ALB	Albumin
2	Hs.654431	NM_000697	ALOX12	Arachidonate 12-lipoxygenase
3	Hs.406238	NM_001159	AOX1	Aldehyde oxidase 1
4	Hs.654439	NM_000041	APOE	Apolipoprotein E
5	Hs.125213	NM_004045	ATOX1	ATX1 antioxidant protein 1 homolog (yeast)
6	Hs.144873	NM_004052	BNIP3	BCL2/adenovirus E1B 19kDa interacting protein 3
7	Hs.502302	NM_001752	CAT	Catalase
8	Hs.514821	NM_002985	CCL5	Chemokine (C-C motif) ligand 5
9	Hs.502917	NM_005125 CCS	CCS	Copper chaperone for superoxide dismutase
10	Hs.292356	NM_000397	CYBB	Cytochrome b-245, beta polypeptide
11	Hs.95120	NM_134268	CYGB	Cytoglobin
12	Hs.498727	NM_014762	DHCR24	24-dehydrocholesterol reductase
13	Hs.272813	NM_175940	DUOX1	Dual oxidase 1
14	Hs.71377	NM_014080	DUOX2	Dual oxidase 2
15	Hs.171695	NM_004417	DUSP1	Dual specificity phosphatase 1
16	Hs.212088	NM_001979	EPHX2	Epoxide hydrolase 2, cytoplasmic
17	Hs.279259	NM_000502	EPX	Eosinophil peroxidase
18	Hs.239	NM_021953	FOXM1	Forkhead box M1
19	Hs.645560	NM_002032	FTH1	Ferritin, heavy polypeptide 1
20	Hs.654465	NM_001498	GCLC	Glutamate-cysteine ligase, catalytic subunit
21	Hs.315562	NM_002061	GCLM	Glutamate-cysteine ligase, modifier subunit
22	Hs.76686	NM_000581	GPX1	Glutathione peroxidase 1
23	Hs.2704	NM_002083	GPX2	Glutathione peroxidase 2 (gastrointestinal)
24	Hs.386793	NM_002084	GPX3	Glutathione peroxidase 3 (plasma)
25	Hs.433951	NM_002085	GPX4	Glutathione peroxidase 4 (phospholipid hydroperoxidase)
26	Hs.248129	NM_001509	GPX5	Glutathione peroxidase 5 (epididymal androgen-related protein)
27	Hs.448570	NM_182701	GPX6	Glutathione peroxidase 6 (olfactory)
28	Hs.43728	NM_015696	GPX7	Glutathione peroxidase 7
29	Hs.271510	NM_000637	GSR	Glutathione reductase
30	Hs.82327	NM_000178	GSS	Glutathione synthetase
31	Hs.523836	NM_000852	GSTP1	Glutathione S-transferase pi 1
32	Hs.655292	NM_001513	GSTZ1	Glutathione transferase zeta 1
33	Hs.520459	NM_001518	GTF2I	General transcription factor IIi
34	Hs.517581	NM_002133	HMOX1	Heme oxygenase (decycling) 1
35	Hs.728810	NM_005345	HSPA1A	Heat shock 70kDa protein 1A
36	Hs.80828	NM_006121	KRT1	Keratin 1
37	Hs.234742	NM_006151	LPO	Lactoperoxidase

38	Hs.517586	NM_005368	MB	Myoglobin
39	Hs.499674	NM_000242	MBL2	Mannose-binding lectin (protein C) 2, soluble
40	Hs.191734	NM_004528	MGST3	Microsomal glutathione S-transferase 3
41	Hs.458272	NM_000250	MPO	Myeloperoxidase
42	Hs.75659	NM_002437	MPV17	MpV17 mitochondrial inner membrane protein
43	Hs.490981	NM_012331	MSRA	Methionine sulfoxide reductase A
44	Hs.73133	NM_005954	MT3	Metallothionein 3
45	Hs.647047	NM_000265	NCF1	Neutrophil cytosolic factor 1
46	Hs.587558	NM_000433	NCF2	Neutrophil cytosolic factor 2
47	Hs.709191	NM_000625	NOS2	Nitric oxide synthase 2, inducible
48	Hs.371036	NM_016931	NOX4	NADPH oxidase 4
49	Hs.657932	NM_024505	NOX5	NADPH oxidase, EF-hand calcium binding domain 5
50	Hs.406515	NM_000903	NQO1	NAD(P)H dehydrogenase, quinone 1
51	Hs.534331	NM_002452	NUDT1	Nudix (nucleoside diphosphate linked moiety X)-type motif 1
52	Hs.148778	NM_181354	OXR1	Oxidation resistance 1
53	Hs.475970	NM_005109	OXSR1	Oxidative-stress responsive 1
54	Hs.368525	NM_020992	PDLIM1	PDZ and LIM domain 1
55	Hs.78016	NM_007254	PNKP	Polynucleotide kinase 3'-phosphatase
56	Hs.180909	NM_002574	PRDX1	Peroxiredoxin 1
57	Hs.432121	NM_005809	PRDX2	Peroxiredoxin 2
58	Hs.523302	NM_006793	PRDX3	Peroxiredoxin 3
59	Hs.83383	NM_006406	PRDX4	Peroxiredoxin 4
60	Hs.502823	NM_181652	PRDX5	Peroxiredoxin 5
61	Hs.120	NM_004905	PRDX6	Peroxiredoxin 6
62	Hs.153310	NM_020820	PREX1	Phosphatidylinositol-3,4,5-trisphosphate-dependent Rac exchange factor 1
63	Hs.472010	NM_183079	PRNP	Prion protein
64	Hs.201978	NM_000962	PTGS1	Prostaglandin-endoperoxide synthase 1 (prostaglandin G/H synthase and cyclooxygenase
65	Hs.196384	NM_000963	PTGS2	Prostaglandin-endoperoxide synthase 2 (prostaglandin G/H synthase and cyclooxygenase
66	Hs.332197	NM_012293	PXDN	Peroxidasin homolog (Drosophila)
67	Hs.134623	NM_014245	RNF7	Ring finger protein 7
68	Hs.128856	NM_182826	SCARA3	Scavenger receptor class A, member 3
69	Hs.32148	NM_203472	SELS	Selenoprotein S
70	Hs.275775	NM_005410	SEPP1	Selenoprotein P, plasma, 1
71	Hs.253495	NM_003019	SFTPD	Surfactant protein D
72	Hs.466693	NM_012237	SIRT2	Sirtuin 2
73	Hs.443914	NM_000454	SOD1	Superoxide dismutase 1, soluble

74	Hs.487046	NM_000636	SOD2	Superoxide dismutase 2, mitochondrial
75	Hs.2420	NM_003102	SOD3	Superoxide dismutase 3, extracellular
76	Hs.437277	NM_003900	SQSTM1	Sequestosome 1
77	Hs.516830	NM_080725	SRXN1	Sulfiredoxin 1
78	Hs.516807	NM_006374	STK25	Serine/threonine kinase 25
79	Hs.467554	NM_000547	TPO	Thyroid peroxidase
80	Hs.134602	NM_003319	TTN	Titin
81	Hs.435136	NM_003329	TXN	Thioredoxin
82	Hs.728817	NM_003330	TXNRD1	Thioredoxin reductase 1
83	Hs.443430	NM_006440	TXNRD2	Thioredoxin reductase 2
84	Hs.80658	NM_003355	UCP2	Uncoupling protein 2 (mitochondrial, proton carrier)
85	Hs.520640	NM_001101	ACTB	Actin, beta
86	Hs.534255	NM_004048	B2M	Beta-2-microglobulin
87	Hs.592355	NM_002046	GAPDH	Glyceraldehyde-3-phosphate dehydrogenase
88	Hs.412707	NM_000194	HPRT1	Hypoxanthine phosphoribosyltransferase 1
89	Hs.546285	NM_001002	RPLP0	Ribosomal protein, large, P0

Genes from 1 to 84 are oxidative stress-related, and from 85 to 89 are housekeeping controls

Table S2 SBSAnalysis of HIF-1a binding site of affected oxidative stress-related gene using gene transcription regulation database (gtrg.biouml.org)

Gene	Effects of hypoxia	Effects of catechin in hypoxia	HIF1a binding site count
ALOX12	Upregulated		10
BNIP3	Upregulated		9
CYBB	Upregulated	Downregulated	0
CYGB	Upregulated		16
DUOX2	Upregulated		7
DUSP1	Upregulated	Downregulated	10
GPX2	Upregulated		2
MBL2	Upregulated		11
NCF2	Upregulated	Downregulated	5
NOX4	Upregulated		7
PTGS1	Upregulated		11
PTGS2	Upregulated	Upregulated	4
SEPP1	Upregulated		Not found
SPINK1	Upregulated		4
TTN	Upregulated		34
DHCR22	Downregulated		Not found
GCLC	Downregulated	Upregulated	38
HPRT1	Downregulated		6
HSPA1A	Downregulated	Upregulated	16
NOX5	Downregulated	Upregulated	26
NUDT1	Downregulated		6
PRNP	Downregulated		11
SRXN1	Downregulated	Downregulated	7
BAG2	Downregulated		9
GCLM	Downregulated		7
GLA	Downregulated		6
HSP90AA1	Downregulated		15
SLC7A11	Downregulated		15
TXNRD1	Downregulated	Upregulated	43
CCL5		Upregulated	0
NCF1		Upregulated	1
PRDX3		Upregulated	5
PRDX4		Upregulated	5
SOD3		Downregulated	2

Date set: meta clusters, Max gene distance: 5000
